# Association between ankylosing spondylitis and m6A methylation

**DOI:** 10.1186/s13018-023-04254-x

**Published:** 2023-10-07

**Authors:** Zhiwei Luan, Yansong Wang

**Affiliations:** 1 Department of Spine Surgery, The First Affiliated Hospital of Harbin Medical University, Harbin, China; 2 NHC Key Laboratory of Cell Transplantation, The First Affiliated Hospital of Harbin Medical University, Harbin, 150001 Heilongjiang Province China; 3 The Key Laboratory of Myocardial Ischemia, Chinese Ministry of Education, Harbin, China

**Keywords:** N6-methyladenosine, Ankylosing spondylitis, Bioinformatics

## Abstract

**Background:**

N6-methyl adenosine (m6A) is the most common reversible mRNA modification in eukaryotes implicated in key roles in various biological processes. The purpose of our analysis was to examine the association of ankylosing spondylitis (AS) with m6A methylation.

**Method:**

We obtained 72 samples from the data set GSE73754, including 52 AS patients and 20 healthy people. We divided the samples into two groups: the experimental group and the control group, and then observed the differences of 26 m6A related genes in the two groups. We also analyzed the correlation between different m6A genes. We used a random forest tree model to screen seven m6A signature genes associated with AS to evaluate its prevalence. Next, the samples were classified according to the m6a content and differential genes. Immune analysis, gene ontology, and KEGG enrichment analyses were performed. Finally, we scored each sample with m6a and analyzed the relationship between different samples and inflammation-related factors.

**Results and conclusion:**

In conclusion, we screened out AS-related genes and the nomogram showed that they were negatively correlated with the incidence of AS. And we found that AS may have some relationship with immunity. Our analysis results could provide further insights into the treatment of AS.

## Introduction

Ankylosing spondylitis (AS), a common clinical type of spondyloarthropathy, is an autoimmune disease characterized by chronic inflammation of axial joints and tendon–ligament–bone attachments. It mainly invades the spine and sacroiliac joints accompanied by multi-system damage. As the disease progresses, patients may experience limited spinal movement, stiffness, and destruction of the hip joint. In severe cases, it can lead to disability and seriously affect the patient's quality of life [1]. The pathogenesis of AS is complex with genetic factors, interactions between microorganisms and the host, differences in hormone levels, imbalance in the intestinal flora, and abnormal immune responses implicated in the process [2–4]. At present, it is believed that disorders of the immune system play drive the occurrence and development of AS. Several studies have shown that both innate and adaptive immune responses are involved in the pathological processes underlying AS.

Similar to DNA methylation, N6-methyl adenosine (m6A) RNA methylation modifications are dynamically reversible and can be catalyzed by methyltransferases (“writers”) or erased by demethylases (“erasers”). Reads are recognized by a set of binding proteins ("readers") with said functions [5–7]. Although the series of processes do not change the principle of complementary base pairing, they functionally (including RNA splicing, transport, and translation) determine the direction of RNA molecules. Thus, m6A methylation modifications are involved in various diseases, especially key in tumor progression [8, 9]. In recent years, studies have shown that m6A methylation regulators are significantly differentially expressed in several malignant tumors, and thus, the influence of abnormal m6A methylation mediated by regulatory factors on various malignant tumors has received extensive attention [10–12]. However, m6A methylation regulators have not been studied in AS, and we intended to elucidate the relationship between m6A methylation and AS.

In this study, we analyzed the expression of m6A methylation regulators in the samples and screened seven disease-related risk genes using the random forest tree model. We observed the relationship of immune cells with immunity in different samples. Our purpose was to examine the correlation between AS and m6A methylation; our findings are expected to provide insight into the diagnosis and treatment of AS in the future.

## Methods

### Data processing and downloading

The GSE73754 dataset was obtained from the Gene Expression Omnibus (GEO) database. A total of 72 samples, including 52 from AS patients and 20 from healthy individuals, were extracted. A total of 26 m6A-related genes including writers (METTL3, METTL14, METTL16, WTAP, VIRMA, ZC3H13, RBM15, RBM15B, CBLL1), readers (YTHDC1, YTHDC2, YTHDF1, YTHDF2, YTHDF3, HNRNPC, FMR1, LRPPRC, HNRNPA2B1, IGFBP1, IGFBP2, IGFBP3, RBMX, ELAVL1, IGF2BP1), and erasers (FTO, ALKBH5) were screened.

### Consensus cluster and immune cell infiltration analyses

In order to comprehensively analyze the relationship between m6A methylation and AS, we used the "ConsensusClusterPlus" package in R to perform a consistent cluster analysis for the patients in the dataset. According to the Cumulative Distribution Function (CDF) curve, stratification was performed based on the optimal “k” value. Principal component analysis (PCA) was used to further confirm the rationality of the consistent cluster-based grouping, and differential analysis for these subtypes was performed. The abundance of immune cells was assessed by single-sample gene set enrichment analysis (ssGSEA) to further investigate correlations, and graphs were plotted to visualize features.

### Random forest model (RF) and support vector machine model (SVM)

We utilized RF and SVM to predict the onset of AS. We built the former using the "RandomForest" package, assessed the importance of each m6A regulator, and screened seven m6A regulators. For SVM, four features were captured, including maximum margin hyperplane, soft margin, kernel function, and separating hyperplane. Each data point was plotted in the n-dimensional space, and a suitable plane was found that well-distinguished the two groups. We again validate these models using inverse cumulative distribution of residuals, box plots of residuals, and receiver operating characteristic (ROC) curves.

### Differential gene expression and functional enrichment between groups

Differentially expressed genes (DEGs) in different clusters were screened using the "limma" package in R, with a significance criterion of P < 0.05. The "clusterprofiler" package in R was employed to perform Gene Ontology (GO) and Kyoto Encyclopedia of Genes and Genomes (KEGG) enrichment analyses for the screened DEGs.

### Calculation of the m6A score

We quantified subtypes by PCA for m6A-based scoring [13]. The formula for the m6A score was as follows: m6A score = PC1i, where PC1 refers to principal component 1 and i represents the level of the corresponding DEG [14].

### Statistical analysis

Statistical analysis was performed using R (version 4.2.0). Linear regression analysis and Pearson correlation coefficient (r) were used to determine the correlation between gene expression patterns. Nonparametric one-way analysis of variance (ANOVA) was used to compare the variables between different groups. The comparison between two groups was made using the t-test. A value of P < 0.05 was indicative of statistical significance.

## Results

### Overview of m6a regulators in AS

**Fig. 1:**
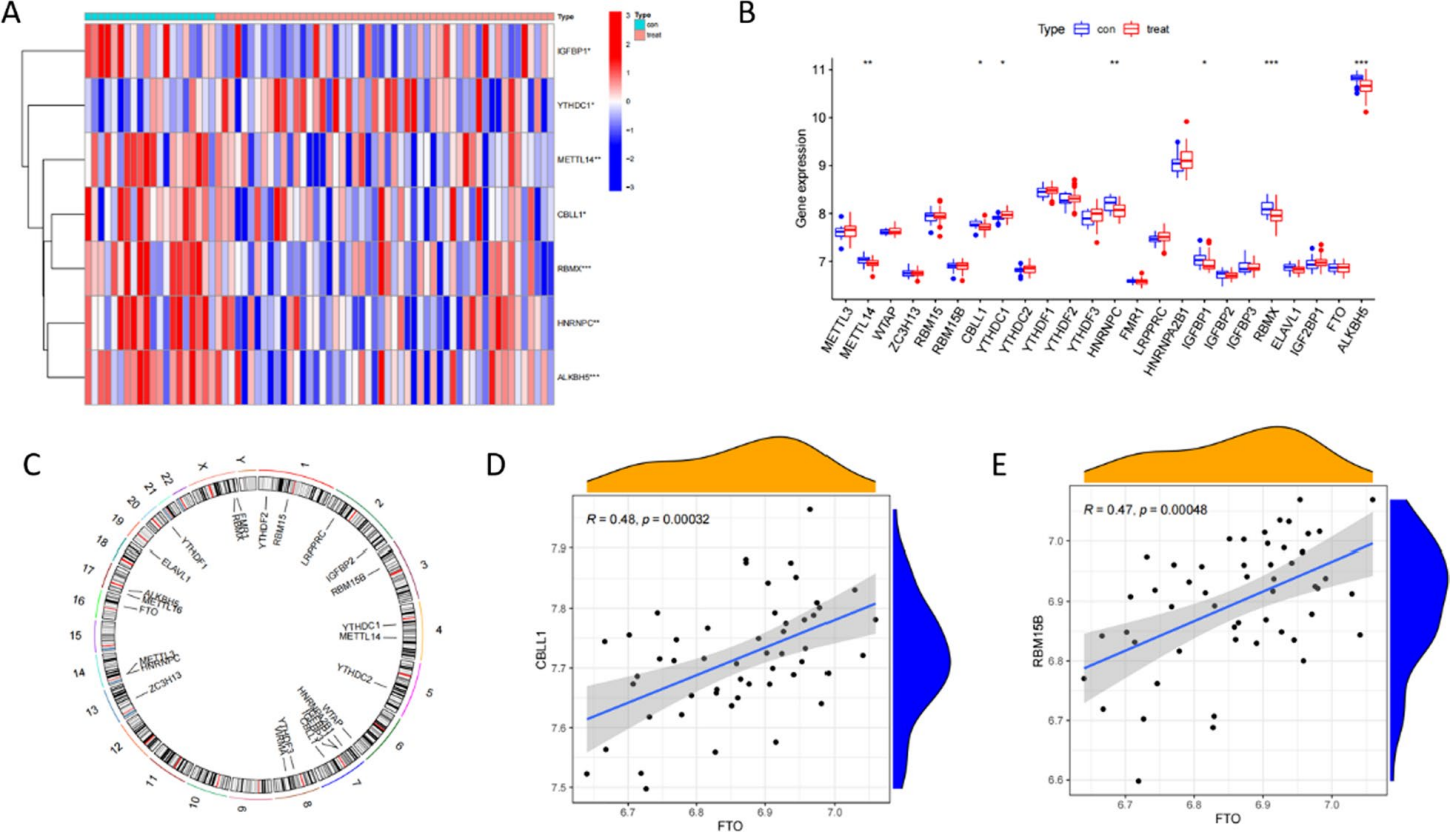
m6a regulators in AS. **A** Heat map of differentially expressed m6a regulators between the two groups. **B** Boxplot of differential expression of 26 m6a regulators between the two groups. **C** Chromosomal location of m6a-related genes. **D** The relationship between FTO and CBLL1. **E** The relationship between FTO and RBM15B

In the GSE73754 dataset, we divided the samples into an experimental group and a control group and observed the differences in the expression of 26 m6A-related genes between them. The expression of seven of the 26 m6a regulators differed significantly between the two groups (Fig. 1A, B). We constructed a circle diagram, and the locations of the 26 m6A-related genes on the chromosomes are shown (Fig. 1C). We also analyzed the associations among different m6A genes and found a certain linear correlation among CBLL1, RBM15B, and FTO, whereby increased expressions of CBLL1 and RBM15B corresponded to high levels of FTO (Fig. 1D, E).

### Selection of the RF and SVM models

**Fig. 2:**
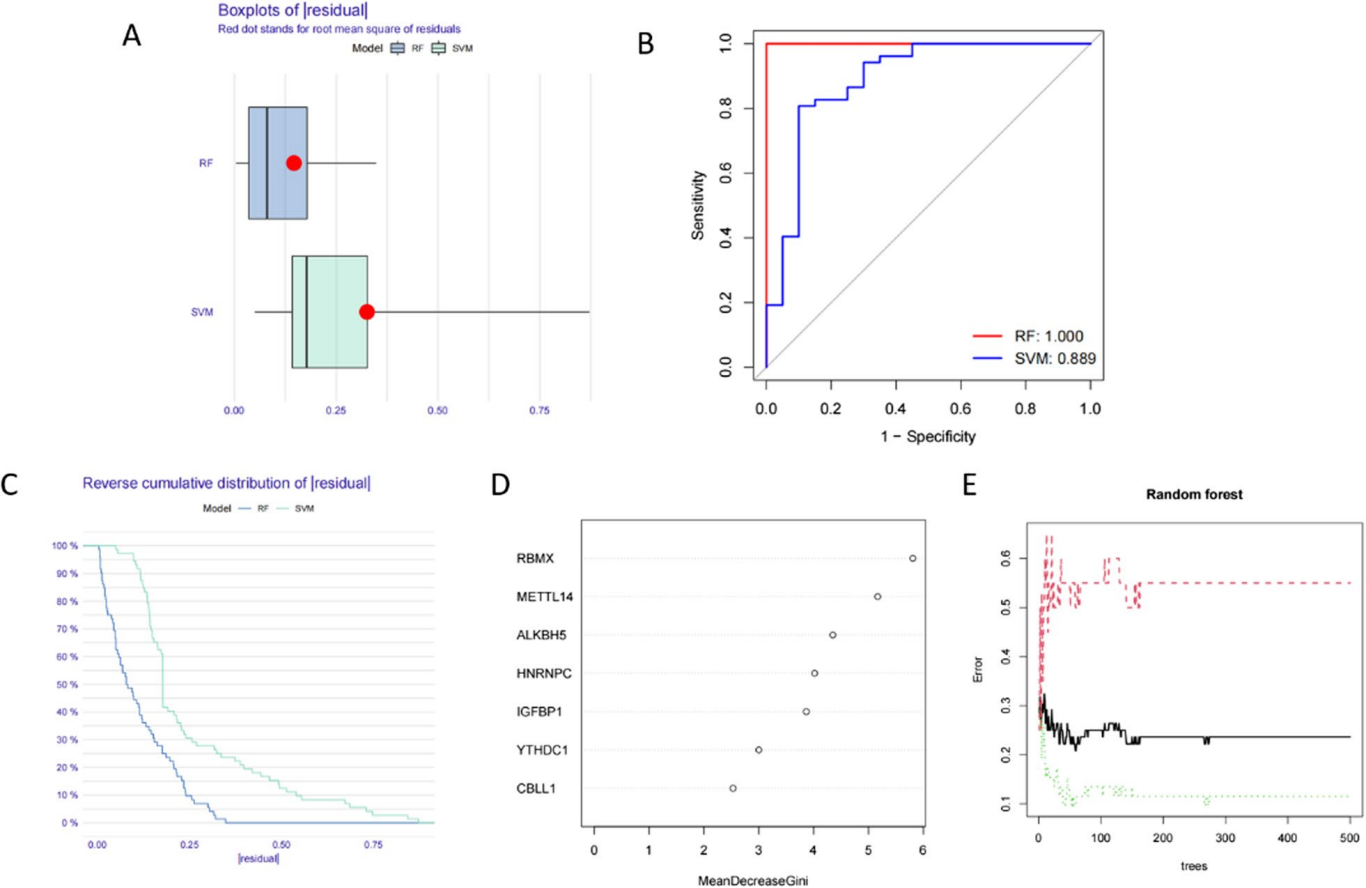
Construction of the RF and SVM models. **A** Boxplots of residual values of RF and SVM models. **B** ROC curves for RF and SVM models. **C** Inverse cumulative distribution plot of residuals of RF and SVM models. **D** Genes in the RF model ranked according to their scores, suggestive of importance; the top three are RBMX, METTL14, and ALKBH5. **E** The influence of the number of decision trees on the error

Next, we compared the RF and SVM models to evaluate the most suitable method for screening disease eigengenes. The residual value of the RF model was lower according to the "inverse cumulative distribution of residuals" and "boxplot of residuals", indicating that it was more suitable for screening AS characteristic genes as compared to the SVM model (Fig. 2 A, C). The area under the ROC curve of the RF model was higher than that of the SVM model, suggesting greater accuracy of the former as compared to the latter. We thus chose the RF model for subsequent analysis (Fig. 2B). Next, we screened AS signature genes using the RF model and found the point with the smallest cross-validation error in the control group, experimental group, and all samples. Subsequently, we found the number of trees corresponding to the point. Ultimately, we identified seven AS signature genes: IGFBP1、YTHDC1、METTL14、CBLL1、RBMX、HNRNPC、ALKBH5. And we scored these based on their importance (Fig. 2D,E).

### Nomogram of AS signature genes

**Fig. 3:**
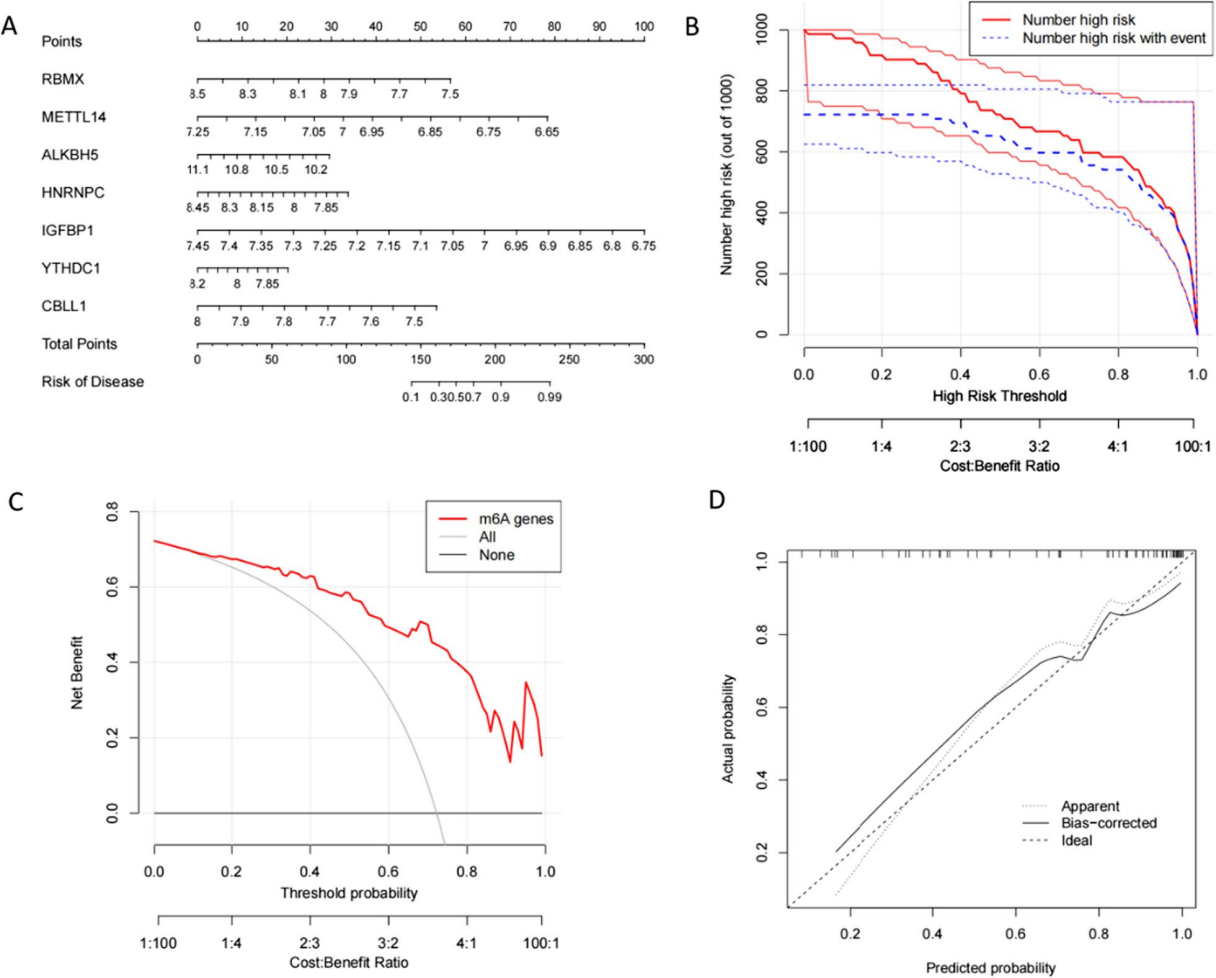
The establishment of the nomogram. **A** Nomogram constructed using seven characteristic genes screened based on the RF model. **B** Clinical impact curve to assess model’s accuracy. **C** Decision curve. The farther the red curve is from the gray curve, the higher the model’s accuracy. **D** Calibration curve. The closer the solid and dashed lines, the more accurate the model is

Next, we constructed a nomogram based on the screened AS characteristic genes. According to the expression of different genes, we calculated the corresponding scores and inferred the incidence of AS according to these scores (Fig. 3A). According to the nomogram, a positive correlation between ALKBH5 and AS, while a negative correlation between RBMX, METTL14, HNRNPC, IGFBP1, YTHDC1, and CBLL1 and AS was observed. The distance between the solid and dashed lines in the calibration curve was relatively close, indicating a substantially high accuracy of the model (Fig. 3D). The decision and clinical impact curves also demonstrated that the constructed nomogram was reliable (Fig. 3 B,C).

### m6a typing

**Fig. 4:**
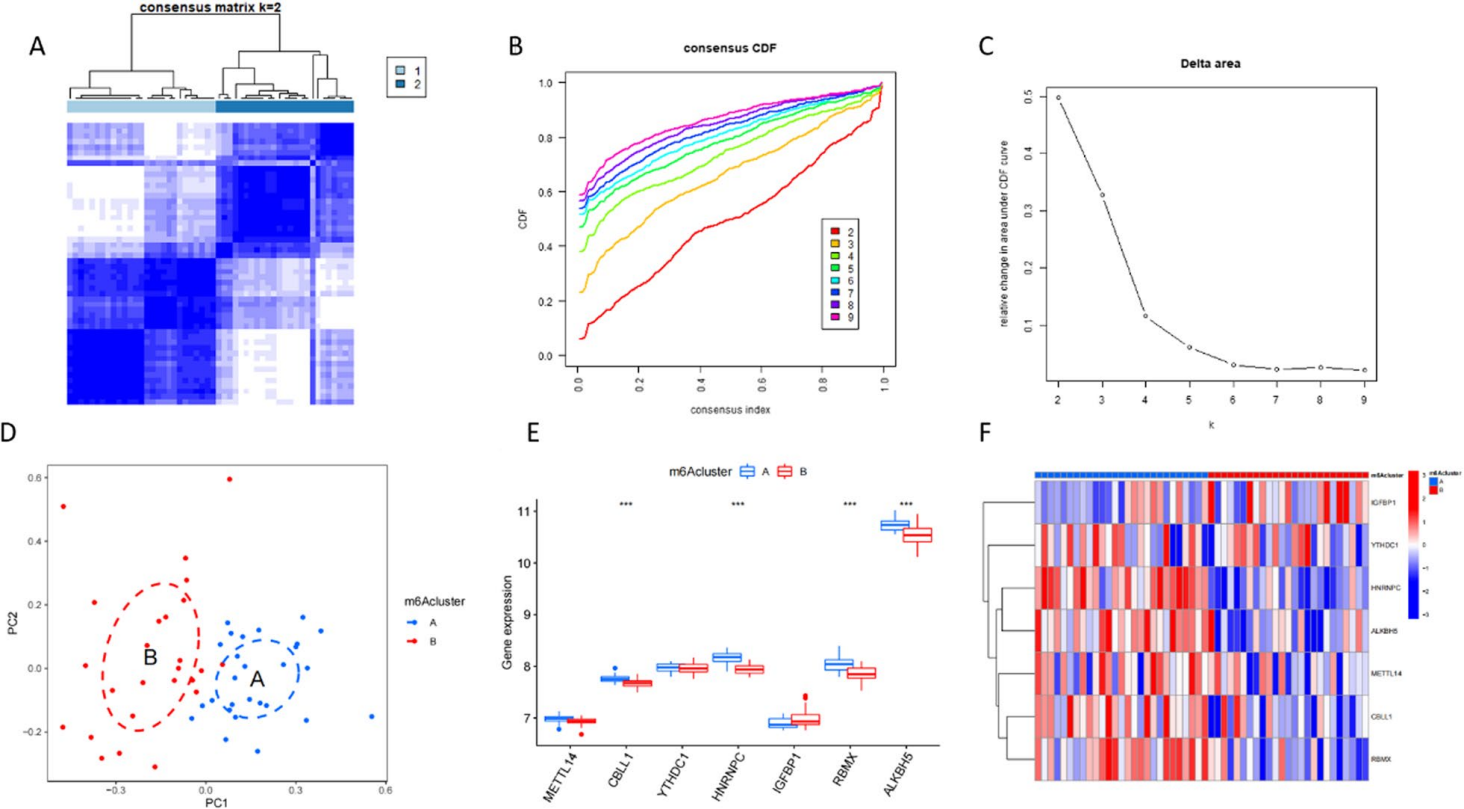
Typing according to m6a-related gene expression. **A** Consensus cluster analysis. **B** CDF curve for consensus clustering. **C** At k = 2, the CDF showed the maximum value. **D** PCA shows a significant difference between clusters A and B. **E** Boxplot showing the differences in m6a signature gene expressions between phenotypes. **F** Heat map of the differences in m6a signature gene expression between different phenotypes

According to differential m6a expressions across samples, we performed a genotyping analysis. At k = 2, the CDF showed the maximum value; thus, we divided all samples into two subtypes (Fig. 4A-C). Subsequent PCA demonstrated that all samples could be distinguished based on the corresponding m6a levels, suggesting that our typing was reliable (Fig. 4D). From the boxplot and gene heat map, the expression of most AS characteristic genes in cluster A was found to be relatively high, while that of the genes in cluster B was relatively low. Significant differences in the expressions of CBLL1, HNRNPC, RBMX, and ALKBH5 were found between the two clusters (Fig. 4E, F).

### Characteristics of m6a typing

**Fig. 5:**
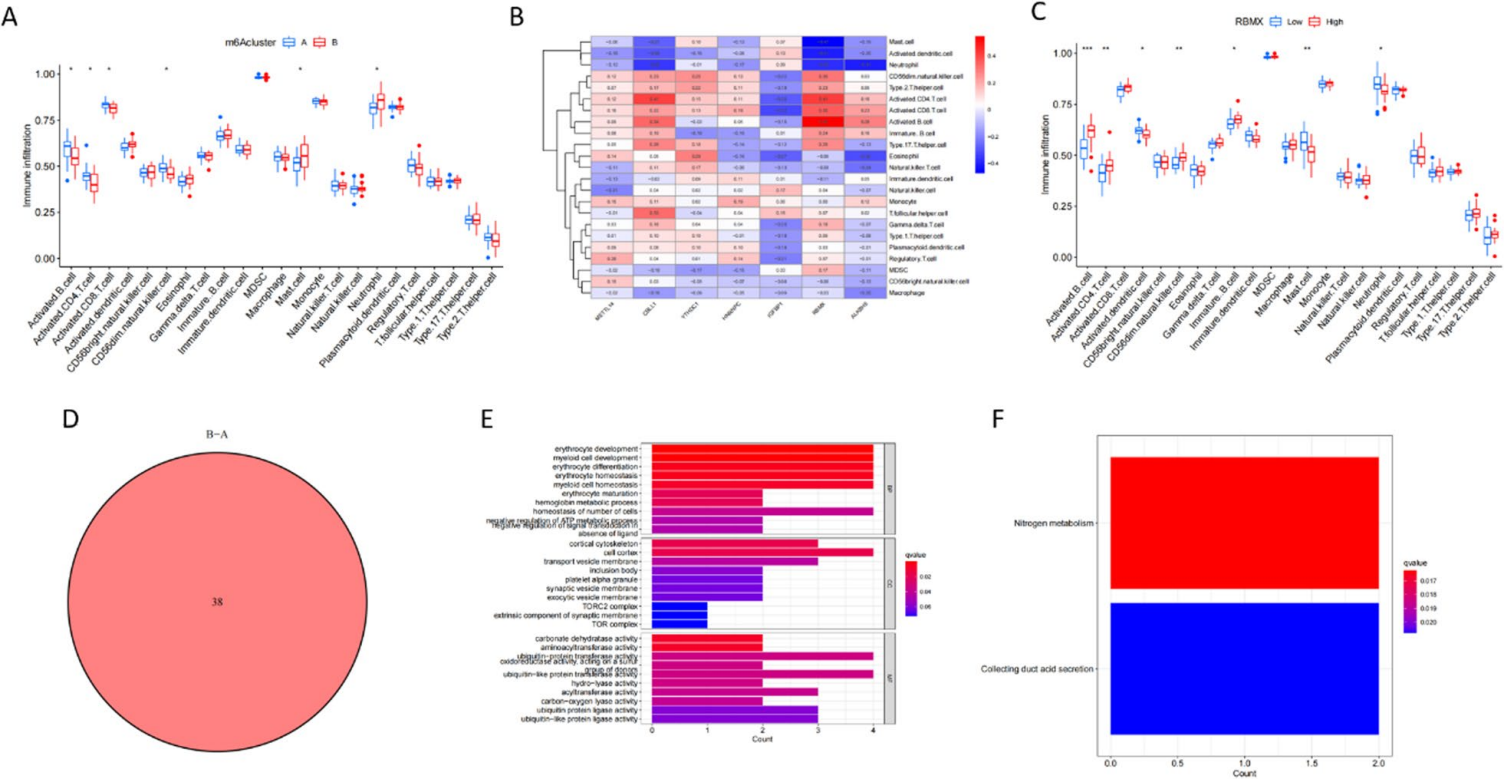
Biological characteristics of m6a typing. **A** Differential analysis for immune cells based on m6a typing. **B** Immune cell correlation analysis for m6a typing; RBMX shows the highest correlation with immune cells. **C** Differential analysis for immune cells between the RBMX high- and RBMX low-expression groups. **D** Venn diagram of DEGs in clusters A and B. **E** GO enrichment analysis for m6a typing. **F** KEGG enrichment analysis for m6a typing

Next, immune cell analysis based on m6a typing was performed to examine the link between m6a gene signature and immunity. Cluster A showed higher infiltration of B-, CD4 T-, CD8 T-, natural killer-, and mast cells relative to cluster B, while the latter showed a higher neutrophil abundance (Fig. 5A). We also analyzed the correlation between m6a signature genes and immune cells. As shown in the figure, RBMX exhibited the highest correlation with immune cells and was used for subsequent analysis (Fig. 5B). The proportions of immature B cells, natural killer cells, CD4T cells, and B cells in the RBMX high-expression group were higher than those in the RBMX low-expression group (Fig. 5C). We identified 38 DEGs between clusters A and B and drew a Venn diagram (Fig. 5D). For these DEGs, we performed GO and KEGG enrichment analyses (Fig. 5E, F). The processes of erythrocyte development, myeloid cell development, and erythrocyte differentiation were enriched. The results of the KEGG enrichment analysis are shown in figure.

### Features of DEG typing

**Fig. 6:**
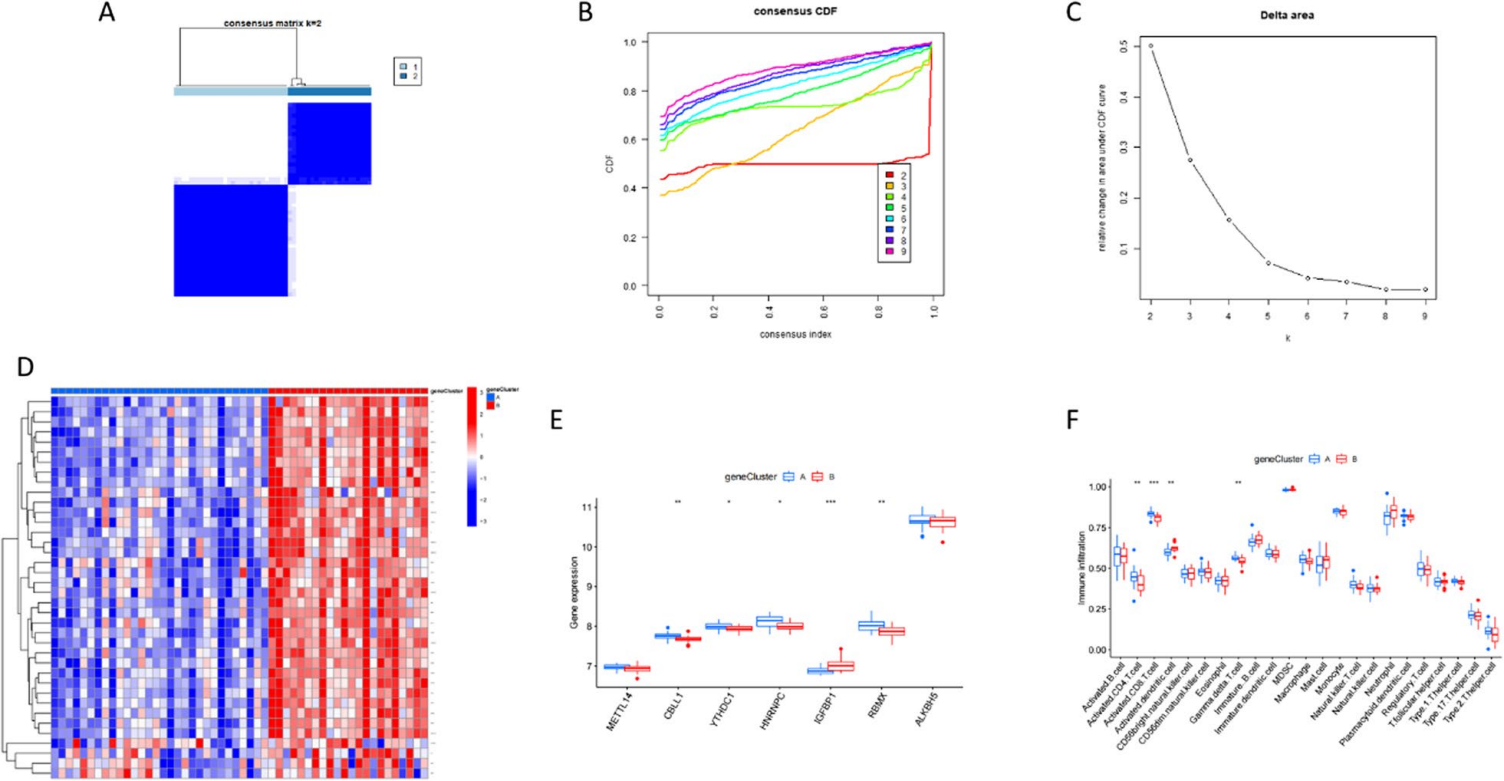
Typing according to DEG levels. **A** Consensus cluster analysis. **B** CDF curve for consensus cluster analysis. **C** At k = 2, the CDF shows the maximum value. **D** The heat map of DEGs between clusters A and B. **E** Boxplots showing differences in m6a signature gene expression between different types. **F** Differences in immune cells between different types

Next, we classified the samples according to DEG levels. At k = 2, CDF showed the maximum value. We, therefore, divided the sample into two groups, whereby the between-group difference was the smallest and the within-group difference was the largest (Fig. 6 A-C). As shown in the heat map, most genes in cluster B show higher expression relative to cluster A (Fig. 6D). m6a differential analysis for genotyping was performed, and most of the m6a signature genes in cluster A showed higher expressions relative to cluster B (Fig. 6E). Finally, we analyzed immune cell proportions between the two clusters. In cluster A, the abundances of CD4 T and CD8 T cells were higher than those in cluster B, while the proportions of dendritic and delta T cells were lower than those in cluster B (Fig. 6F).

### m6a score-based signature and inflammatory factors

**Fig. 7:**
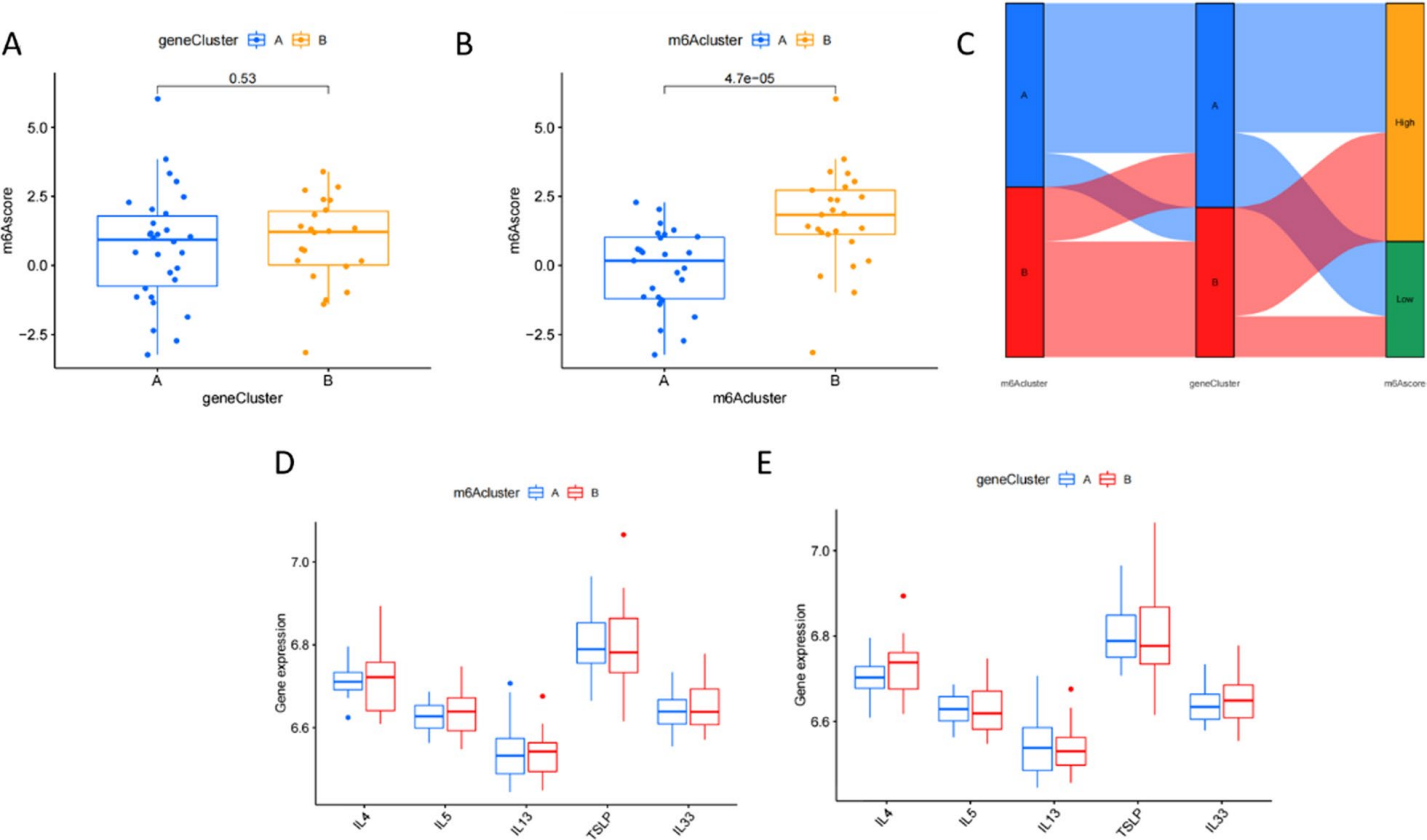
m6a scores for different types. **A** Differences in m6a scores of genotypes. **B** Differences in m6a scores for m6a types. **C** Sankey diagram for different types and m6a scores. **D** Differences in the expression of inflammatory factors based on m6a typing. **E** Differences in the expression of inflammatory factors based on gene typing

PCA is an efficient statistical analysis technique to quantify m6a signatures. Based on the results of our analysis, m6a cluster A showed a lower score than m6a cluster B, while there were no differences between the scores of gene clusters A and B (Fig. 7A, B). In the Sankey diagram, a certain similarity between the results of m6a typing and genotyping was observed (Fig. 7C). Inflammatory factors and immunity are closely related. Finally, we analyzed the relationship between different types and inflammatory factors. The data showed no differences in the expression of inflammatory factors among subtypes (Fig. 7D, E).

## Discussion

AS is a chronic autoimmune disease [15], the etiology of which remains clear. Currently, it is believed that AS is related to genetic, infection, immune, and endocrine factors [16–18]. AS involves the spine, sacroiliac joints, hip joints, and other bones, and can also include tendons and ligaments around the joints, resulting in ossification and ankylosis of these structures [19, 20]. The disease causes physical dysfunction in patients and simultaneous serious psychological problems, including loss of confidence in life, resulting in a heavy burden to the family and society [21, 22]. As an important modification in the transcriptome, m6a methylation affects the stability, splicing, and translation of mRNA, thus playing an important role in post-transcriptional regulation. Previous studies show that regulation of m6A methylation modification is closely related to the occurrence and development of tumors via tumor-promoting effects through several tumor-related molecules or signaling pathways [23]. Methylation of m6A can affect the progression of osteosarcoma by affecting tumor cell proliferation, apoptosis, invasion ability, and migration ability [24]. And m6A methylation can have a certain impact on osteoporosis by regulating osteoblast differentiation and regulating osteoclast bone resorption function [25, 26]. However, the relationship between m6a methylation and AS is unknown. In this study, we used the RF model to construct a nomogram to estimate the incidence of AS; we hypothesized that m6a methylation may be related to AS.

We screened seven m6a characteristic genes using the RF model, namely ALKBH5, RBMX, METTL14, HNRNPC, IGFBP1, YTHDC1, and CBLL1. Among them, three genes, RBMX, METTL14, and ALKBH5, showed the highest scores, suggestive of their importance. RBMX, a heterogeneous ribonucleoprotein, is mainly localized in the nucleus and consists of 391 amino acid residues with an RNA-binding domain at the amino terminus of the protein [27, 28]. This structural feature reveals the physical association of RBMX with RNA, and the binding region is required for RNA processing and metabolism [29]. Therefore, the level of RBMX expression and its biological activity may affect the overall gene expression in cells to maintain normal cellular homeostasis [21]. Recent studies have implicated RBMX protein expression in apoptosis [30]. Previous studies show that all X-chromosome-located RBMX genes are significantly up-regulated in breast cancer and correlate positively with the expression of the pro-apoptotic gene, BAX [31]. Based on these previous findings, RBMX plays an important role in apoptosis or programmed cell death, which may be related to AS. Recently, m6A modification mediated by METTL14 has been implicated in the occurrence and development of tumors, and METTL14 plays a role in promoting cancer. For example, m6A methylation levels are significantly higher in approximately 70% of pancreatic cancer samples, and dysregulation of METTL14 can affect m6A levels in pancreatic cancer cells [32]. The expression of METTL14 is also up-regulated in breast cancer tissues, thus promoting breast cancer cell migration and invasion by regulating the expression of hsa-miR-146a-5p [33]. ALKBH5 demethylation plays an important role in various life processes, especially its complex role in tumors, which is worthy of further discussion. ALKBH5 can stabilize the expression of the target gene FOXM1 mRNA by reducing the levels of m6A, thereby inducing the occurrence of glioblastoma [33]. LKBH5 can also enhance the stability of BCL-2 mRNA through demethylation and promote the proliferation and invasion of ovarian cancer cells [34]. Thus, RBMX, METTL14, and ALKBH5, the three m6a characteristic genes in this study, may be related to AS. The nomogram constructed showed a negative correlation of these genes with the incidence of AS. Immune correlation analysis showed the association of these three genes with immunity, especially RBMX, which may underlie their importance in AS.

AS is an autoimmune disease. Several inflammatory T cells, monocytes, and macrophages infiltrate bones, joints, and synovial tissues of AS patients, causing progressive inflammation. Significantly increased levels of alpha globulin, gamma globulin, immunoglobulin IgG, IgA, IgM, serum complement C3, and complement C4 expressions, and Thl/Th2 and Thl7/Treg ratios have been reported in patients with AS [35, 36]. The pathogenesis of AS is related to the abnormal function of B cells. The proportion of CD19 + B cells in AS patients with active phase or peripheral joint involvement was higher than that in patients with stable phase or axial joint involvement, and the proportion of CD19 + B cells in active AS patients after 12 weeks of etanercept treatment did not decrease [37]. Another study showed that the levels of expression of BAFF and its receptor, BR3, in peripheral blood leukocytes of AS patients were significantly up-regulated, and the two were significantly positively correlated; the abundance of CD19 + CD38 + antibody-secreting cells also increased [38]. Our data suggested that both m6a cluster A and gene cluster A had greater immune infiltration relative to m6a cluster B and gene cluster B, which may be due to AS prevalence.

In conclusion, we assessed the relationship between m6a methylation and AS, identified the characteristic genes of m6a, and discussed their relationship with immunity. Our findings may provide strategies for the treatment of AS in the future.
